# *rac*-{[2-(Diphenyl­thio­phosphor­yl)ferrocen­yl]meth­yl}dimethyl­ammonium diphenyl­dithio­phosphinate

**DOI:** 10.1107/S1600536812009129

**Published:** 2012-03-07

**Authors:** Nardjes Mouas, Hocine Merazig, Jean-Claude Daran, Eric Manoury

**Affiliations:** a Unité de Recherche de Chimie Moléculaire et Structurale CHEMS, Université Mentouri, Constantine, Algeria; b CNRS, LCC, 205 route de Narbonne, BP 44099, F-31077, Toulouse cedex 4, France

## Abstract

2-(Diphenyl­thio­phosphino)dimethyl­amino­methyl­ferrocene is a key inter­mediate in the synthesis of various ferrocenyl ligands. During one such synthesis, the title compound, [Fe(C_5_H_5_)(C_20_H_22_NPS)](C_12_H_10_PS_2_), was isolated as a by-product. It is built up by association of (2-(diphenyl­phosphino)ferrocen­yl)meth­yl)dimethyl­ammonium cations and diphenyl­phosphino dithio­ate anions. N—H⋯S, C—H⋯S and C—H⋯π inter­actions link the anions and cations. Each anion–cation pair is linked two by two through C—H⋯π inter­actions, forming pseudo dimers.

## Related literature

For the synthesis of various ferrocenyl ligands, see: Audin *et al.* (2010 ▶); Le Roux *et al.* (2007 ▶); Routaboul *et al.* (2005 ▶, 2007 ▶). For related structures containing the C_12_H_10_PS_2_ anion, see: Alison *et al.* (1971 ▶); Fackler *et al.* (1982 ▶); Silvestru *et al.* (1995 ▶). For related ferrocenyl ammonium structures, see: Štěpnička & Císařová, (2003 ▶). For a related ferrocenyl­amine structure, see: Mateus *et al.* (2006 ▶).
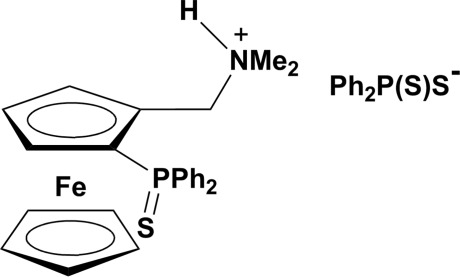


## Experimental

### 

#### Crystal data

[Fe(C_5_H_5_)(C_20_H_22_NPS)](C_12_H_10_PS_2_)
*M* *_r_* = 709.65
Monoclinic, 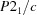

*a* = 14.7800 (3) Å
*b* = 18.3770 (3) Å
*c* = 13.6318 (3) Å
β = 112.557 (2)°
*V* = 3419.31 (12) Å^3^
*Z* = 4
Mo *K*α radiation
μ = 0.75 mm^−1^
*T* = 180 K
0.38 × 0.13 × 0.06 mm

#### Data collection

Agilent Xcalibur Eos Gemini ultra diffractometer
Absorption correction: multi-scan (*SCALE3 ABSPACK* in *CrysAlis PRO*; Agilent, 2011 ▶) *T*_min_ = 0.815, *T*_max_ = 1.000
37299 measured reflections
7498 independent reflections
6435 reflections with *I* > 2σ(*I*)
*R*_int_ = 0.032

#### Refinement

*R*[*F* ^2^ > 2σ(*F* ^2^)] = 0.031
*wR*(*F* ^2^) = 0.079
*S* = 1.05
7498 reflections
402 parameters
H atoms treated by a mixture of independent and constrained refinement
Δρ_max_ = 0.49 e Å^−3^
Δρ_min_ = −0.27 e Å^−3^

### 

Data collection: *CrysAlis PRO* (Agilent, 2011 ▶); cell refinement: *CrysAlis PRO*; data reduction: *CrysAlis PRO*; program(s) used to solve structure: *SIR97* (Altomare *et al.*, 1999 ▶); program(s) used to refine structure: *SHELXL97* (Sheldrick, 2008 ▶); molecular graphics: *ORTEPIII* (Burnett & Johnson, 1996 ▶) and *ORTEP-3 for Windows* (Farrugia, 1997 ▶); software used to prepare material for publication: *SHELXL97* and *PLATON* (Spek, 2009) ▶.

## Supplementary Material

Crystal structure: contains datablock(s) I, global. DOI: 10.1107/S1600536812009129/hp2031sup1.cif

Structure factors: contains datablock(s) I. DOI: 10.1107/S1600536812009129/hp2031Isup2.hkl

Additional supplementary materials:  crystallographic information; 3D view; checkCIF report

## Figures and Tables

**Table 1:** Hydrogen-bond geometry (Å, °) *Cg*1 and *Cg*2 are the centroids of the C111–C116 and C221–C226 phenyl rings, respectively.

*D*—H⋯*A*	*D*—H	H⋯*A*	*D*⋯*A*	*D*—H⋯*A*
N1—H1⋯S21	0.85 (2)	2.34 (2)	3.1516 (15)	160.3 (19)
C21—H21*B*⋯S1	0.99	2.87	3.664 (2)	137
C22—H22*C*⋯*Cg*2^i^	0.99	2.75	3.621 (3)	149
C23—H23*A*⋯*Cg*1	0.99	2.75	3.483 (2)	132

Symmetry code: (i) 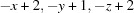.

**Fig. 1.**
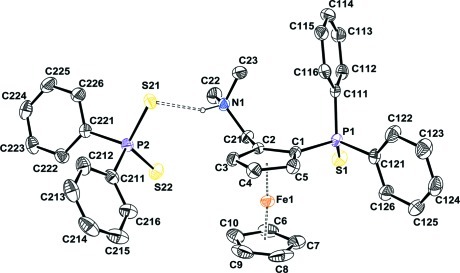
The asymmetric unit of (I) with the atom-labelling scheme. Displacement ellipsoids are drawn at the 50% probability level. H atom is represented as small sphere of arbitrary radii. Hydrogen bond is shown as dashed lines. H atoms not involved in hydrogen bondings within the Figure have been omitted for clarity.

**Fig. 2.**
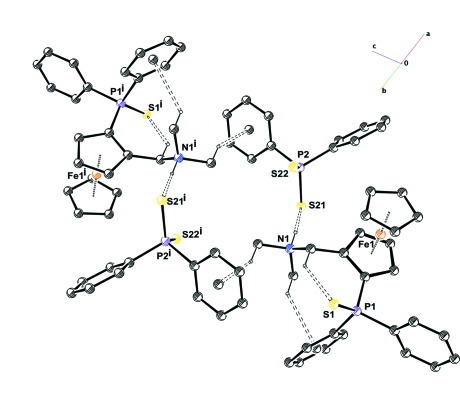
View showing the formation of pseudo dimer resulting from C—H···π interaction. H atoms are represented as small sphere of arbitrary radii and the H bonds are shown as dashed lines. H atoms not involved in hydrogen bonding have been omitted for clarity. [Cg1 and Cg2 are respectively the centroids of the C111 to C116 and C221 to C226 phenyl ring]. [Symmetry code: (i) -x + 2, -y + 1, -z + 2]

## supplementary crystallographic information

### Comment

2-(diphenylthiophosphino)dimethylaminomethylferrocene is a key intermediate in the synthesis of various ferrocenyl ligands (Routaboul *et al.*, 2005; Mateus *et al.*, 2006; Routaboul *et al.*, 2007; Le Roux *et al.*, 2007; Audin *et al.*, 2010;), in our laboratories. The last step of the synthesis of 2-(diphenylthiophosphino)dimethylaminomethylferrocene is a sulfurization of 2-(diphenylphosphino)dimethylaminomethylferrocene without any purification with an excess of elemental sulfur (Mateus *et al.*, 2006). During this synthesis, small amounts of dimethyl-(2-(diphenylthiophosphino)ferrocenyl)methylammonium diphenylphosphinodithioato can be observed in the crude materials. We were able to obtain pure salt fractions by flash chromatography on silicagel. Monocrystals suitable for X-ray diffraction analysis could be grown from a dichloromethane solution by slow diffusion of hexane.

The asymmetric unit of the title compound contains a (2-(diphenylphosphino)ferrocenyl)methyl)dimethylammonium cation and a diphenylphosphino dithioate anion which are linked through N—H···S hydrogen bond (Fig. 1; Table 1). Besides this rather strong hydrogen bond, there are weaker C—H···S and C—H···π hydrogen interactions. The anion-cation couple are linked two by two through intermolecular C—H···π interactions (Fig. 2; Table 1).

In the cation, the two Cp rings have roughly a staggered conformation with a twist angle of 20.6 (2)° and they are slightly bent with respect to each other making a dihedral angle of 4.72 (12)°. As observed in the related 2-(diphenylthiophosphino)-dimethylaminomethylferrocene (Mateus *et al.*, 2006), the S atom is displaced *undo* towards the Fe atom by 1.149 (4) Å from the Cp ring. The C2—C21—N1 plane is making a dihedral angle of 58.9 (1)° with the corresponding Cp ring whereas in the above ferrocenylamine (Mateus *et al.*, 2006) the corresponding angle was roughly 90°. The C21—N1 distance of 1.502 (2) Å is similar to the 1.526 (2) Å observed in the reported ferrocenylammonium cation, [FeCp_2_PPh_2_(CH_2_NMe_2_CH_2_Ph)]^+^ (Štěpnička & Císařová, 2003).

The geometry of the anion fully agrees with related structures containing the same anion (Alison *et al.*, 1971; Fackler *et al.*, 1982; Silvestru *et al.*, 1995).

### Experimental

In a schlenk tube under argon 4gr of crude (2-diphenylphosphino) dimethylaminomethyl ferrocene (0,47 mmol) were dissolved in 100 ml of dichloromethane, 1,7gr of sulfur (53 mmol) were then added and the solution was heated to reflux for 2 h. The crude product was purified and crystallized at RT. Several days later, orange crystals suitable for X-ray analyses, were obtained.

### Refinement

All H atoms attached to C atoms were fixed geometrically and treated as riding with C—H = 0.98 Å (methyl), 0.99 Å (methylene) and 0.95 Å (aromatic) with *U*_iso_(H) = 1.2*U*_eq_(C_methylene_, C_aromatic_) and *U*_iso_(H) = 1.5*U*_eq_(C_methyl_). H atom attached to nitrogen was freely refined with *U*_iso_(H) = 1.2*U*_eq_(N).

### Crystal data

**Table:**

[Fe(C_5_H_5_)(C_20_H_22_NPS)](C_12_H_10_PS_2_)	*F*(000) = 1480
*M**_r_* = 709.65	*D*_x_ = 1.379 Mg m^−^^3^
Monoclinic, *P*2_1_/*c*	Mo *K*α radiation, λ = 0.71073 Å
Hall symbol: -P 2ybc	Cell parameters from 11690 reflections
*a* = 14.7800 (3) Å	θ = 3.0–29.1°
*b* = 18.3770 (3) Å	µ = 0.75 mm^−^^1^
*c* = 13.6318 (3) Å	*T* = 180 K
β = 112.557 (2)°	Box, brown
*V* = 3419.31 (12) Å^3^	0.38 × 0.13 × 0.06 mm
*Z* = 4	

### Data collection

**Table:**

Agilent Xcalibur Eos Gemini ultra diffractometer	7498 independent reflections
Radiation source: Enhance (Mo) X-ray Source	6435 reflections with *I* > 2σ(*I*)
Graphite monochromator	*R*_int_ = 0.032
Detector resolution: 16.1978 pixels mm^-1^	θ_max_ = 27.1°, θ_min_ = 3.0°
ω scans	*h* = −18→18
Absorption correction: multi-scan (SCALE3 ABSPACK in *CrysAlis PRO*; Agilent, 2011)	*k* = −23→23
*T*_min_ = 0.815, *T*_max_ = 1.000	*l* = −17→16
37299 measured reflections	

### Refinement

**Table:**

Refinement on *F*^2^	Primary atom site location: structure-invariant direct methods
Least-squares matrix: full	Secondary atom site location: difference Fourier map
*R*[*F*^2^ > 2σ(*F*^2^)] = 0.031	Hydrogen site location: inferred from neighbouring sites
*wR*(*F*^2^) = 0.079	H atoms treated by a mixture of independent and constrained refinement
*S* = 1.05	*w* = 1/[σ^2^(*F*_o_^2^) + (0.0316*P*)^2^ + 2.1534*P*] where *P* = (*F*_o_^2^ + 2*F*_c_^2^)/3
7498 reflections	(Δ/σ)_max_ = 0.001
402 parameters	Δρ_max_ = 0.49 e Å^−^^3^
0 restraints	Δρ_min_ = −0.27 e Å^−^^3^

### Special details

**Table:**

Geometry. All e.s.d.'s (except the e.s.d. in the dihedral angle between two l.s. planes) are estimated using the full covariance matrix. The cell e.s.d.'s are taken into account individually in the estimation of e.s.d.'s in distances, angles and torsion angles; correlations between e.s.d.'s in cell parameters are only used when they are defined by crystal symmetry. An approximate (isotropic) treatment of cell e.s.d.'s is used for estimating e.s.d.'s involving l.s. planes.
Refinement. Refinement of *F*^2^ against ALL reflections. The weighted *R*-factor *wR* and goodness of fit *S* are based on *F*^2^, conventional *R*-factors *R* are based on *F*, with *F* set to zero for negative *F*^2^. The threshold expression of *F*^2^ > σ(*F*^2^) is used only for calculating *R*-factors(gt) *etc*. and is not relevant to the choice of reflections for refinement. *R*-factors based on *F*^2^ are statistically about twice as large as those based on *F*, and *R*- factors based on ALL data will be even larger.

### Fractional atomic coordinates and isotropic or equivalent isotropic displacement parameters (Å^2^)

**Table:**

	*x*	*y*	*z*	*U*_iso_*/*U*_eq_	
Fe1	0.892126 (18)	0.295821 (13)	0.56329 (2)	0.02385 (7)	
S1	0.62046 (3)	0.37182 (3)	0.53349 (4)	0.03057 (11)	
P1	0.69099 (3)	0.40945 (2)	0.44871 (3)	0.02035 (10)	
N1	0.92524 (11)	0.49481 (8)	0.74750 (11)	0.0221 (3)	
H1	0.9858 (16)	0.4861 (11)	0.7672 (16)	0.027*	
C1	0.81988 (12)	0.38914 (9)	0.49855 (13)	0.0199 (3)	
C2	0.89066 (12)	0.40119 (9)	0.60503 (13)	0.0203 (3)	
C3	0.98477 (12)	0.38249 (10)	0.60566 (14)	0.0251 (4)	
H3	1.0451	0.3854	0.6653	0.030*	
C4	0.97303 (13)	0.35884 (10)	0.50256 (15)	0.0278 (4)	
H4	1.0241	0.3430	0.4815	0.033*	
C5	0.87217 (12)	0.36281 (10)	0.43610 (14)	0.0251 (4)	
H5	0.8441	0.3502	0.3630	0.030*	
C6	0.83827 (17)	0.23364 (11)	0.65368 (19)	0.0417 (5)	
H6	0.8017	0.2508	0.6931	0.050*	
C7	0.79979 (18)	0.20995 (12)	0.5482 (2)	0.0491 (6)	
H7	0.7323	0.2083	0.5030	0.059*	
C8	0.8793 (2)	0.18870 (12)	0.5202 (2)	0.0552 (7)	
H8	0.8744	0.1700	0.4534	0.066*	
C9	0.96609 (18)	0.20020 (12)	0.6086 (2)	0.0491 (6)	
H9	1.0306	0.1911	0.6124	0.059*	
C10	0.94077 (17)	0.22754 (12)	0.69090 (19)	0.0445 (5)	
H10	0.9855	0.2399	0.7602	0.053*	
C21	0.87113 (13)	0.42619 (9)	0.69984 (13)	0.0233 (4)	
H21A	0.8908	0.3873	0.7543	0.028*	
H21B	0.7999	0.4345	0.6784	0.028*	
C22	0.90524 (15)	0.51676 (12)	0.84249 (15)	0.0342 (4)	
H22A	0.9459	0.5588	0.8764	0.051*	
H22B	0.9207	0.4762	0.8929	0.051*	
H22C	0.8359	0.5297	0.8206	0.051*	
C23	0.90543 (15)	0.55545 (10)	0.67055 (15)	0.0333 (4)	
H23A	0.8359	0.5685	0.6448	0.050*	
H23B	0.9219	0.5403	0.6105	0.050*	
H23C	0.9454	0.5976	0.7054	0.050*	
C111	0.69061 (12)	0.50804 (9)	0.44182 (12)	0.0207 (3)	
C112	0.63090 (13)	0.54821 (10)	0.48002 (14)	0.0269 (4)	
H112	0.5868	0.5238	0.5045	0.032*	
C113	0.63565 (14)	0.62385 (11)	0.48240 (15)	0.0330 (4)	
H113	0.5949	0.6510	0.5084	0.040*	
C114	0.69980 (15)	0.65936 (11)	0.44694 (15)	0.0336 (4)	
H114	0.7040	0.7110	0.4500	0.040*	
C115	0.75816 (13)	0.61995 (10)	0.40692 (14)	0.0288 (4)	
H115	0.8008	0.6447	0.3809	0.035*	
C116	0.75428 (12)	0.54460 (10)	0.40491 (13)	0.0243 (4)	
H116	0.7950	0.5177	0.3784	0.029*	
C121	0.64309 (12)	0.37339 (10)	0.31452 (13)	0.0240 (4)	
C122	0.63419 (15)	0.41465 (12)	0.22647 (15)	0.0356 (5)	
H122	0.6536	0.4643	0.2346	0.043*	
C123	0.59665 (17)	0.38328 (13)	0.12585 (16)	0.0437 (5)	
H123	0.5905	0.4117	0.0654	0.052*	
C124	0.56839 (15)	0.31161 (13)	0.11324 (16)	0.0390 (5)	
H124	0.5427	0.2907	0.0443	0.047*	
C125	0.57726 (16)	0.27025 (12)	0.20011 (16)	0.0410 (5)	
H125	0.5582	0.2205	0.1914	0.049*	
C126	0.61405 (15)	0.30098 (11)	0.30073 (15)	0.0350 (4)	
H126	0.6194	0.2723	0.3607	0.042*	
P2	1.19253 (3)	0.42161 (3)	0.91598 (3)	0.02193 (10)	
S21	1.15395 (3)	0.50508 (3)	0.81403 (4)	0.02754 (10)	
S22	1.08593 (3)	0.36503 (3)	0.93493 (4)	0.03142 (11)	
C211	1.26918 (13)	0.36027 (10)	0.87503 (13)	0.0263 (4)	
C212	1.35891 (14)	0.38428 (12)	0.87664 (15)	0.0333 (4)	
H212	1.3819	0.4316	0.9022	0.040*	
C213	1.41491 (15)	0.33888 (14)	0.84076 (16)	0.0422 (5)	
H213	1.4762	0.3553	0.8417	0.051*	
C214	1.38169 (17)	0.26997 (13)	0.80366 (17)	0.0452 (6)	
H214	1.4204	0.2390	0.7795	0.054*	
C215	1.29285 (18)	0.24609 (12)	0.80160 (18)	0.0445 (5)	
H215	1.2699	0.1988	0.7755	0.053*	
C216	1.23679 (16)	0.29114 (11)	0.83768 (16)	0.0346 (4)	
H216	1.1757	0.2744	0.8367	0.042*	
C221	1.27607 (12)	0.45558 (10)	1.04489 (13)	0.0227 (3)	
C222	1.32713 (14)	0.40705 (11)	1.12579 (15)	0.0310 (4)	
H222	1.3217	0.3562	1.1124	0.037*	
C223	1.38586 (15)	0.43272 (12)	1.22579 (15)	0.0369 (5)	
H223	1.4207	0.3994	1.2806	0.044*	
C224	1.39368 (15)	0.50654 (13)	1.24572 (16)	0.0392 (5)	
H224	1.4336	0.5240	1.3143	0.047*	
C225	1.34355 (16)	0.55488 (12)	1.16604 (17)	0.0411 (5)	
H225	1.3486	0.6057	1.1800	0.049*	
C226	1.28564 (14)	0.52961 (11)	1.06545 (15)	0.0324 (4)	
H226	1.2524	0.5632	1.0105	0.039*	

### Atomic displacement parameters (Å^2^)

**Table:**

	*U*^11^	*U*^22^	*U*^33^	*U*^12^	*U*^13^	*U*^23^
Fe1	0.02534 (13)	0.01906 (13)	0.02753 (14)	0.00148 (10)	0.01055 (10)	−0.00295 (10)
S1	0.0301 (2)	0.0363 (3)	0.0316 (2)	−0.00764 (19)	0.0189 (2)	−0.0016 (2)
P1	0.0183 (2)	0.0243 (2)	0.0188 (2)	−0.00269 (16)	0.00753 (16)	−0.00222 (17)
N1	0.0195 (7)	0.0237 (8)	0.0208 (7)	0.0004 (6)	0.0052 (6)	−0.0052 (6)
C1	0.0196 (8)	0.0202 (8)	0.0193 (8)	−0.0019 (6)	0.0066 (6)	−0.0009 (6)
C2	0.0205 (8)	0.0175 (8)	0.0216 (8)	−0.0003 (6)	0.0065 (6)	−0.0012 (6)
C3	0.0207 (8)	0.0263 (9)	0.0261 (9)	−0.0017 (7)	0.0065 (7)	−0.0037 (7)
C4	0.0232 (9)	0.0320 (10)	0.0320 (9)	−0.0003 (7)	0.0147 (7)	−0.0034 (8)
C5	0.0242 (8)	0.0298 (10)	0.0228 (8)	−0.0024 (7)	0.0105 (7)	−0.0039 (7)
C6	0.0528 (13)	0.0252 (10)	0.0573 (14)	0.0024 (9)	0.0323 (11)	0.0078 (10)
C7	0.0445 (13)	0.0244 (11)	0.0664 (16)	−0.0087 (9)	0.0081 (11)	0.0080 (10)
C8	0.095 (2)	0.0198 (11)	0.0596 (15)	−0.0031 (11)	0.0390 (15)	−0.0102 (10)
C9	0.0495 (13)	0.0270 (11)	0.0766 (17)	0.0154 (10)	0.0308 (13)	0.0101 (11)
C10	0.0504 (13)	0.0305 (11)	0.0454 (13)	0.0038 (10)	0.0104 (10)	0.0131 (10)
C21	0.0243 (8)	0.0238 (9)	0.0216 (8)	−0.0033 (7)	0.0086 (7)	−0.0035 (7)
C22	0.0364 (10)	0.0397 (12)	0.0281 (10)	0.0009 (9)	0.0141 (8)	−0.0110 (8)
C23	0.0406 (11)	0.0225 (9)	0.0299 (10)	−0.0012 (8)	0.0059 (8)	−0.0013 (8)
C111	0.0172 (7)	0.0249 (9)	0.0168 (8)	−0.0005 (6)	0.0028 (6)	−0.0013 (6)
C112	0.0204 (8)	0.0344 (10)	0.0260 (9)	0.0007 (7)	0.0089 (7)	−0.0031 (8)
C113	0.0310 (10)	0.0329 (11)	0.0337 (10)	0.0072 (8)	0.0110 (8)	−0.0047 (8)
C114	0.0400 (11)	0.0246 (10)	0.0298 (10)	0.0038 (8)	0.0063 (8)	0.0006 (8)
C115	0.0294 (9)	0.0301 (10)	0.0239 (9)	−0.0017 (8)	0.0070 (7)	0.0051 (8)
C116	0.0225 (8)	0.0294 (10)	0.0198 (8)	0.0017 (7)	0.0069 (7)	0.0016 (7)
C121	0.0175 (8)	0.0314 (10)	0.0222 (8)	−0.0013 (7)	0.0066 (7)	−0.0038 (7)
C122	0.0423 (11)	0.0375 (11)	0.0255 (9)	−0.0113 (9)	0.0113 (8)	−0.0036 (8)
C123	0.0531 (13)	0.0522 (14)	0.0232 (10)	−0.0094 (11)	0.0116 (9)	−0.0021 (9)
C124	0.0370 (11)	0.0500 (13)	0.0253 (10)	−0.0004 (9)	0.0067 (8)	−0.0122 (9)
C125	0.0469 (12)	0.0334 (11)	0.0346 (11)	−0.0028 (9)	0.0065 (9)	−0.0112 (9)
C126	0.0419 (11)	0.0319 (11)	0.0253 (9)	−0.0036 (9)	0.0062 (8)	−0.0035 (8)
P2	0.0210 (2)	0.0259 (2)	0.0184 (2)	0.00091 (17)	0.00710 (17)	−0.00071 (17)
S21	0.0231 (2)	0.0321 (2)	0.0252 (2)	0.00201 (17)	0.00693 (17)	0.00576 (18)
S22	0.0290 (2)	0.0373 (3)	0.0304 (2)	−0.00756 (19)	0.01418 (19)	−0.0032 (2)
C211	0.0293 (9)	0.0309 (10)	0.0186 (8)	0.0077 (7)	0.0092 (7)	0.0034 (7)
C212	0.0287 (9)	0.0449 (12)	0.0258 (9)	0.0048 (8)	0.0099 (8)	−0.0001 (8)
C213	0.0302 (10)	0.0665 (16)	0.0306 (10)	0.0127 (10)	0.0125 (8)	0.0051 (10)
C214	0.0535 (14)	0.0518 (14)	0.0335 (11)	0.0283 (11)	0.0203 (10)	0.0081 (10)
C215	0.0653 (15)	0.0314 (11)	0.0420 (12)	0.0142 (10)	0.0262 (11)	0.0040 (10)
C216	0.0433 (11)	0.0297 (10)	0.0348 (10)	0.0054 (8)	0.0194 (9)	0.0023 (8)
C221	0.0208 (8)	0.0279 (9)	0.0208 (8)	0.0004 (7)	0.0094 (7)	−0.0005 (7)
C222	0.0329 (10)	0.0311 (10)	0.0261 (9)	0.0041 (8)	0.0083 (8)	0.0011 (8)
C223	0.0311 (10)	0.0492 (13)	0.0233 (9)	0.0060 (9)	0.0025 (8)	0.0026 (9)
C224	0.0309 (10)	0.0545 (14)	0.0273 (10)	−0.0090 (9)	0.0056 (8)	−0.0116 (9)
C225	0.0453 (12)	0.0336 (11)	0.0393 (12)	−0.0096 (9)	0.0105 (10)	−0.0101 (9)
C226	0.0347 (10)	0.0292 (10)	0.0297 (10)	−0.0028 (8)	0.0083 (8)	−0.0005 (8)

### Geometric parameters (Å, º)

**Table:**

Fe1—C2	2.0208 (17)	C112—C113	1.391 (3)
Fe1—C1	2.0340 (17)	C112—H112	0.9500
Fe1—C3	2.0344 (18)	C113—C114	1.382 (3)
Fe1—C9	2.036 (2)	C113—H113	0.9500
Fe1—C10	2.039 (2)	C114—C115	1.388 (3)
Fe1—C8	2.042 (2)	C114—H114	0.9500
Fe1—C7	2.044 (2)	C115—C116	1.386 (3)
Fe1—C6	2.052 (2)	C115—H115	0.9500
Fe1—C5	2.0533 (18)	C116—H116	0.9500
Fe1—C4	2.0532 (18)	C121—C122	1.383 (3)
S1—P1	1.9557 (6)	C121—C126	1.389 (3)
P1—C1	1.7992 (17)	C122—C123	1.392 (3)
P1—C111	1.8141 (18)	C122—H122	0.9500
P1—C121	1.8146 (17)	C123—C124	1.372 (3)
N1—C23	1.481 (2)	C123—H123	0.9500
N1—C22	1.490 (2)	C124—C125	1.370 (3)
N1—C21	1.501 (2)	C124—H124	0.9500
N1—H1	0.85 (2)	C125—C126	1.387 (3)
C1—C5	1.436 (2)	C125—H125	0.9500
C1—C2	1.444 (2)	C126—H126	0.9500
C2—C3	1.430 (2)	P2—C221	1.8270 (17)
C2—C21	1.500 (2)	P2—C211	1.8305 (18)
C3—C4	1.417 (2)	P2—S22	1.9856 (6)
C3—H3	0.9500	P2—S21	2.0004 (6)
C4—C5	1.419 (2)	C211—C216	1.384 (3)
C4—H4	0.9500	C211—C212	1.390 (3)
C5—H5	0.9500	C212—C213	1.390 (3)
C6—C7	1.398 (3)	C212—H212	0.9500
C6—C10	1.405 (3)	C213—C214	1.382 (3)
C6—H6	0.9500	C213—H213	0.9500
C7—C8	1.422 (4)	C214—C215	1.374 (3)
C7—H7	0.9500	C214—H214	0.9500
C8—C9	1.399 (4)	C215—C216	1.388 (3)
C8—H8	0.9500	C215—H215	0.9500
C9—C10	1.404 (3)	C216—H216	0.9500
C9—H9	0.9500	C221—C226	1.385 (3)
C10—H10	0.9500	C221—C222	1.394 (3)
C21—H21A	0.9900	C222—C223	1.388 (3)
C21—H21B	0.9900	C222—H222	0.9500
C22—H22A	0.9800	C223—C224	1.380 (3)
C22—H22B	0.9800	C223—H223	0.9500
C22—H22C	0.9800	C224—C225	1.379 (3)
C23—H23A	0.9800	C224—H224	0.9500
C23—H23B	0.9800	C225—C226	1.388 (3)
C23—H23C	0.9800	C225—H225	0.9500
C111—C112	1.396 (2)	C226—H226	0.9500
C111—C116	1.398 (2)		
			
C2—Fe1—C1	41.73 (6)	C10—C9—Fe1	69.96 (12)
C2—Fe1—C3	41.28 (7)	C8—C9—H9	126.1
C1—Fe1—C3	69.34 (7)	C10—C9—H9	126.1
C2—Fe1—C9	143.50 (9)	Fe1—C9—H9	125.3
C1—Fe1—C9	172.17 (9)	C9—C10—C6	108.7 (2)
C3—Fe1—C9	111.40 (9)	C9—C10—Fe1	69.74 (13)
C2—Fe1—C10	112.82 (8)	C6—C10—Fe1	70.40 (12)
C1—Fe1—C10	147.52 (8)	C9—C10—H10	125.6
C3—Fe1—C10	105.12 (9)	C6—C10—H10	125.6
C9—Fe1—C10	40.31 (10)	Fe1—C10—H10	125.8
C2—Fe1—C8	174.18 (10)	C2—C21—N1	112.54 (14)
C1—Fe1—C8	135.46 (10)	C2—C21—H21A	109.1
C3—Fe1—C8	144.53 (10)	N1—C21—H21A	109.1
C9—Fe1—C8	40.11 (11)	C2—C21—H21B	109.1
C10—Fe1—C8	67.38 (10)	N1—C21—H21B	109.1
C2—Fe1—C7	133.57 (9)	H21A—C21—H21B	107.8
C1—Fe1—C7	112.83 (8)	N1—C22—H22A	109.5
C3—Fe1—C7	169.60 (9)	N1—C22—H22B	109.5
C9—Fe1—C7	67.94 (10)	H22A—C22—H22B	109.5
C10—Fe1—C7	67.32 (10)	N1—C22—H22C	109.5
C8—Fe1—C7	40.72 (11)	H22A—C22—H22C	109.5
C2—Fe1—C6	108.42 (8)	H22B—C22—H22C	109.5
C1—Fe1—C6	118.04 (8)	N1—C23—H23A	109.5
C3—Fe1—C6	129.75 (9)	N1—C23—H23B	109.5
C9—Fe1—C6	67.90 (9)	H23A—C23—H23B	109.5
C10—Fe1—C6	40.19 (9)	N1—C23—H23C	109.5
C8—Fe1—C6	67.73 (10)	H23A—C23—H23C	109.5
C7—Fe1—C6	39.91 (10)	H23B—C23—H23C	109.5
C2—Fe1—C5	69.49 (7)	C112—C111—C116	119.31 (17)
C1—Fe1—C5	41.12 (7)	C112—C111—P1	119.92 (13)
C3—Fe1—C5	68.49 (7)	C116—C111—P1	120.64 (13)
C9—Fe1—C5	131.32 (9)	C113—C112—C111	120.24 (17)
C10—Fe1—C5	168.16 (8)	C113—C112—H112	119.9
C8—Fe1—C5	111.59 (9)	C111—C112—H112	119.9
C7—Fe1—C5	120.23 (9)	C114—C113—C112	119.92 (18)
C6—Fe1—C5	151.38 (8)	C114—C113—H113	120.0
C2—Fe1—C4	69.14 (7)	C112—C113—H113	120.0
C1—Fe1—C4	68.87 (7)	C113—C114—C115	120.27 (18)
C3—Fe1—C4	40.56 (7)	C113—C114—H114	119.9
C9—Fe1—C4	106.34 (9)	C115—C114—H114	119.9
C10—Fe1—C4	128.44 (9)	C116—C115—C114	120.12 (18)
C8—Fe1—C4	115.60 (9)	C116—C115—H115	119.9
C7—Fe1—C4	149.79 (9)	C114—C115—H115	119.9
C6—Fe1—C4	167.51 (9)	C115—C116—C111	120.11 (17)
C5—Fe1—C4	40.42 (7)	C115—C116—H116	119.9
C1—P1—C111	102.03 (8)	C111—C116—H116	119.9
C1—P1—C121	104.60 (8)	C122—C121—C126	119.11 (17)
C111—P1—C121	108.68 (8)	C122—C121—P1	123.04 (14)
C1—P1—S1	115.51 (6)	C126—C121—P1	117.85 (14)
C111—P1—S1	113.07 (6)	C121—C122—C123	119.84 (19)
C121—P1—S1	112.11 (6)	C121—C122—H122	120.1
C23—N1—C22	111.15 (15)	C123—C122—H122	120.1
C23—N1—C21	113.45 (13)	C124—C123—C122	120.5 (2)
C22—N1—C21	110.67 (14)	C124—C123—H123	119.7
C23—N1—H1	105.4 (14)	C122—C123—H123	119.7
C22—N1—H1	108.3 (14)	C125—C124—C123	120.03 (19)
C21—N1—H1	107.5 (14)	C125—C124—H124	120.0
C5—C1—C2	107.49 (14)	C123—C124—H124	120.0
C5—C1—P1	125.37 (13)	C124—C125—C126	120.0 (2)
C2—C1—P1	126.99 (12)	C124—C125—H125	120.0
C5—C1—Fe1	70.16 (10)	C126—C125—H125	120.0
C2—C1—Fe1	68.65 (9)	C125—C126—C121	120.50 (19)
P1—C1—Fe1	129.96 (9)	C125—C126—H126	119.7
C3—C2—C1	107.29 (14)	C121—C126—H126	119.7
C3—C2—C21	125.22 (15)	C221—P2—C211	103.57 (8)
C1—C2—C21	127.46 (15)	C221—P2—S22	109.33 (6)
C3—C2—Fe1	69.87 (10)	C211—P2—S22	109.40 (7)
C1—C2—Fe1	69.63 (9)	C221—P2—S21	108.27 (6)
C21—C2—Fe1	124.22 (12)	C211—P2—S21	107.77 (6)
C4—C3—C2	108.61 (15)	S22—P2—S21	117.58 (3)
C4—C3—Fe1	70.43 (10)	C216—C211—C212	119.37 (18)
C2—C3—Fe1	68.85 (10)	C216—C211—P2	120.67 (15)
C4—C3—H3	125.7	C212—C211—P2	119.88 (15)
C2—C3—H3	125.7	C213—C212—C211	119.8 (2)
Fe1—C3—H3	126.6	C213—C212—H212	120.1
C3—C4—C5	108.46 (15)	C211—C212—H212	120.1
C3—C4—Fe1	69.01 (10)	C214—C213—C212	120.2 (2)
C5—C4—Fe1	69.80 (10)	C214—C213—H213	119.9
C3—C4—H4	125.8	C212—C213—H213	119.9
C5—C4—H4	125.8	C215—C214—C213	120.2 (2)
Fe1—C4—H4	127.0	C215—C214—H214	119.9
C4—C5—C1	108.15 (15)	C213—C214—H214	119.9
C4—C5—Fe1	69.79 (11)	C214—C215—C216	119.8 (2)
C1—C5—Fe1	68.72 (10)	C214—C215—H215	120.1
C4—C5—H5	125.9	C216—C215—H215	120.1
C1—C5—H5	125.9	C211—C216—C215	120.6 (2)
Fe1—C5—H5	127.1	C211—C216—H216	119.7
C7—C6—C10	107.7 (2)	C215—C216—H216	119.7
C7—C6—Fe1	69.75 (13)	C226—C221—C222	119.09 (17)
C10—C6—Fe1	69.42 (12)	C226—C221—P2	120.61 (14)
C7—C6—H6	126.2	C222—C221—P2	120.22 (14)
C10—C6—H6	126.2	C223—C222—C221	120.29 (19)
Fe1—C6—H6	126.2	C223—C222—H222	119.9
C6—C7—C8	108.0 (2)	C221—C222—H222	119.9
C6—C7—Fe1	70.33 (12)	C224—C223—C222	120.08 (19)
C8—C7—Fe1	69.55 (13)	C224—C223—H223	120.0
C6—C7—H7	126.0	C222—C223—H223	120.0
C8—C7—H7	126.0	C225—C224—C223	119.96 (19)
Fe1—C7—H7	125.7	C225—C224—H224	120.0
C9—C8—C7	107.9 (2)	C223—C224—H224	120.0
C9—C8—Fe1	69.72 (13)	C224—C225—C226	120.3 (2)
C7—C8—Fe1	69.73 (12)	C224—C225—H225	119.9
C9—C8—H8	126.1	C226—C225—H225	119.9
C7—C8—H8	126.1	C221—C226—C225	120.31 (19)
Fe1—C8—H8	126.1	C221—C226—H226	119.8
C8—C9—C10	107.8 (2)	C225—C226—H226	119.8
C8—C9—Fe1	70.17 (13)		

### Hydrogen-bond geometry (Å, º)

*Cg*1 and *Cg*2 are the centroids of the C111–C116 and C221–C226 phenyl rings, respectively.

**Table:**

*D*—H···*A*	*D*—H	H···*A*	*D*···*A*	*D*—H···*A*
N1—H1···S21	0.85 (2)	2.34 (2)	3.1516 (15)	160.3 (19)
C21—H21*B*···S1	0.99	2.87	3.664 (2)	137
C22—H22*C*···*Cg*2^i^	0.99	2.75	3.621 (3)	149
C23—H23*A*···*Cg*1	0.99	2.75	3.483 (2)	132

Symmetry code: (i) −*x*+2, −*y*+1, −*z*+2.
