# *rac*-{[2-(Diphenyl­thio­phosphor­yl)ferrocen­yl]meth­yl}dimethyl­ammonium diphenyl­dithio­phosphinate. Corrigendum

**DOI:** 10.1107/S2056989020011986

**Published:** 2020-09-30

**Authors:** Toma Nardjes Mouas, Hocine Merazig, Jean-Claude Daran, Eric Manoury

**Affiliations:** a Unité de Recherche de Chimie Moléculaire et Structurale CHEMS, Université Mentouri, Constantine, Algeria; b CNRS, LCC, 205 route de Narbonne, BP 44099, F-31077, Toulouse cedex 4, France

## Abstract

In the paper by Mouas *et al.* [*Acta Cryst.* (2012), E**68**, m381–m382], there was an error in the name of the first author.

## Abstract 2

Corrigendum to *Acta Cryst.* (2012), E**68**, m381–m382.

The name of the first author in the paper by Mouas *et al.* (2012 ▸) is incorrect and should be ‘Toma Nardjes Mouas’, as given above.
